# Research on the Measurement of Particulate Matter Concentration in Diesel Vehicle Exhaust Using the Light Scattering Method

**DOI:** 10.3390/s25061898

**Published:** 2025-03-18

**Authors:** Jie Wang, Xinjian Liu, Chao Wang, Yiyang Qiu, Jie Zhou, Qi Dang

**Affiliations:** 1 School of Automotive and Traffic Engineering, Wuhan University of Science and Technology, Wuhan 430070, China; wangjie1980@wust.edu.cn (J.W.); xinjian3002@outlook.com (X.L.); mr_qyy@163.com (Y.Q.); 2 Huayuan Electric Power Design Institute, Wuhan 430050, China; summer330@163.com; 3 Wuhan Bitzer Technology Co., Ltd., Wuhan 430050, China; dangqi@aliyun.com

**Keywords:** scattering, exhaust, particulate matter concentration, anti-contamination, sensor

## Abstract

To address the current issues with diesel vehicle exhaust after-treatment system particulate sensors—such as low accuracy and inability to perform continuous measurements of particulate mass concentration—a new sensor based on the light scattering method is proposed. During the research, it was found that the light scattering method can be affected by soot particles in the exhaust, which contaminate the optical components and reduces measurement accuracy. To solve this issue, a structure with alumina ceramic embedded lenses and optical fibers was designed, effectively improving the sensor’s resistance to contamination. The detection device is based on the principle of light scattering, and a particulate concentration measurement system with a 90° scattering angle was built. Calibration experiments were conducted using the dust particles generated by the device. The experimental results show that this sensor can measure particulate concentrations accurately, in real time, and with good stability, achieving a calibration error of less than ±5%.

## 1. Introduction

Carbon peak and carbon neutrality are among the core issues of China’s modernization [1]. Synergistically advancing pollution reduction and carbon emission reduction has become an inevitable choice for the comprehensive green transformation of China’s economic and social development. To prevent environmental pollution from diesel vehicles and improve air quality, the National VI emission standards have set stricter limits on pollutant emissions from heavy-duty diesel vehicles, with nitrogen oxides and particulate matter (PM) emission limits reduced by 77% and 67% compared to the National V standards [2]. The diesel particulate filter (DPF) is one of the most effective systems for reducing PM emissions outside the engine, with a filtration efficiency of over 95%. The timing of DPF regeneration and the assessment of its health status are directly related to the concentration of PM inside it [3]. Currently, PM sensors used for diesel vehicle emissions have issues with low precision and the inability to measure continuously. Therefore, there is an urgent need for a high-precision PM monitoring device that can be used at the rear end of diesel particulate filters for real-time and continuous measurement.

Currently, the most widely used PM sensors are resistive-type sensors developed jointly by Bosch (Germany) and NTK (Japan). These sensors use multi-layer ceramic technology to convert the resistance value into particulate concentration [4,5]. However, because the resistance value is cumulative, it cannot provide real-time particulate concentration data [6]. In 2016, Hagen and colleagues introduced a new capacitive-type PM sensor, which improved the sensitivity and response time for detecting particulates [7]. Nevertheless, both resistive and capacitive PM sensors still cannot meet the demands for high accuracy and real-time continuous measurement. As a result, many researchers have shifted their focus to optical methods for measuring PM concentration [8]. In 2009, X. Wang and colleagues developed an optical instrument for measuring particulate mass concentration (0.001~150 mg/m^3^). This instrument, which combines photometric and light pulse measurements, can estimate aerosol mass concentrations higher than those measured by typical single-particle counters. This technology, in collaboration with TSI, a U.S. particulate sensor manufacturer, contributed to the development of the particulate sensor market [9]. In 2010, Renard and colleagues designed an online monitor for atmospheric particulate mass concentration using forward small-angle light scattering. This instrument can measure particulate mass concentration for different particle sizes but cannot distinguish the composition of the particles [10]. In 2012, Jourdainne and colleagues developed a flexible and compact optical scattering-based PM concentration sensor that can perform online measurements of particulate mass concentration [11]. In 2016, Gao and colleagues designed a lightweight, highly sensitive PM2.5 mass concentration measurement instrument, which had a small measurement error [12]. In 2020, Wang’s research team at the Desert Research Institute in the U.S. evaluated emission data models for air pollution caused by vehicle exhaust, biomass burning in transportation, and household cooking with biomass fuel in Beijing. They also predicted the emission prospects for new synthetic liquid fuels [13].

Over the years, light-scattering sensors have demonstrated significant advantages in measuring particulate matter (PM) concentration. These sensors are compact, portable, and easy to maintain and operate, which reduces manufacturing costs. Additionally, they exhibit high sensitivity to particles of various sizes, enabling precise measurement of extremely small particles. However, light-scattering sensors can sometimes be affected by particle deposition that contaminates optical components, such as PM accumulating within the optical cavity of diesel exhaust pipes, which can interfere with light scattering and transmission, thereby reducing measurement accuracy. To overcome this limitation, this study proposes a novel design that integrates light scattering with high-temperature-resistant ceramic plates, resulting in a high-precision PM concentration detection sensor capable of real-time, continuous measurement and resistance to contamination.

## 2. Principles and Design of Light Scattering Particle Matter Sensor

### 2.1. Principles of Measurement and Theoretical Fundamentals

There are two primary methods utilized for evaluating the mass concentration of particulate matter based on the principle of light scattering: the photometer method and the single-particle counting method [14]. In the photometer method, under constant parameters of the particles, the intensity of scattered light is directly proportional to the mass concentration. Hence, the mass concentration of particles is determinable through the measurement of the scattered light flux produced by the particle set at various spatial angles, along with the application of the calibration conversion factor [15]. While this method effectively measures PM2.5 in specific circumstances and exhibits strong measurement linearity across a broad spectrum, discrepancies arise when the particulate characteristics within the monitoring environment vary significantly from those present in the calibration particles. Consequently, the readings may not accurately depict the actual mass concentration of particles within the monitoring environment. Hence, recalibration of the instrument utilizing particles with characteristics akin to those in the monitoring setting is essential to ascertain its conversion factor [16]. The photometer method lacks the capability to determine particle size. When sampling, a cutting device is necessary to eliminate particles larger than 2.5 μm from the sample. The single-particle light scattering method, also known as the optical particle counter (OPC) method, operates differently from the photometer method. In this method, particles are individually allowed to pass through the laser photosensitive area, producing a scattered pulse signal corresponding to each single particle. The size of each pulse signal corresponds to the particle size, while the number of pulses corresponds to the quantity of particles. Hence, by measuring the particle light scattering pulse signal and with knowledge of the calibration curve of pulse voltage and particle size, PN values for various particle size segments can be determined. Subsequently, the mass concentration of the particle is calculated using the PN-PM mass concentration conversion algorithm [17].

**Figure 1:**
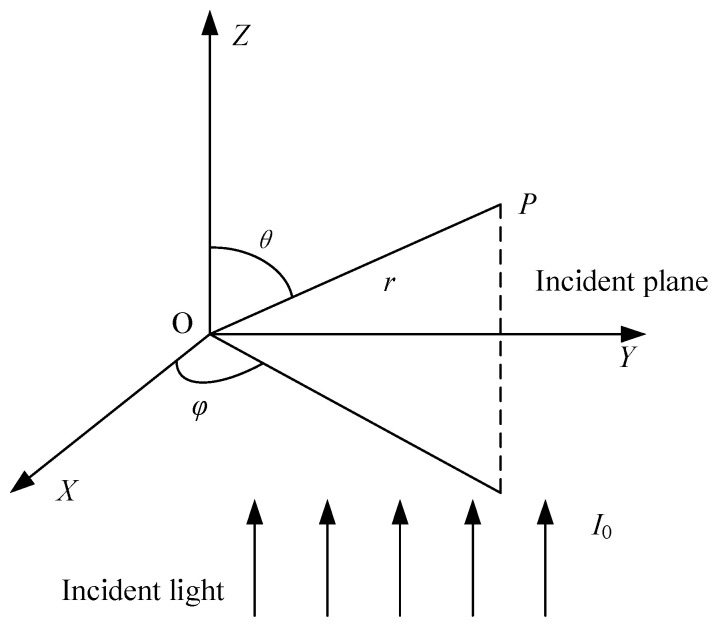
Schematic diagram of Mie scattering by a spherical particle.

Mie scattering is the theoretical basis for measuring particle mass concentration using the single-particle light scattering method [18]. The scattering diagram and the scattering light intensity are shown in Figure 1 and Equation (1), respectively. In Figure 1, P is the observation point.

(1) $$I_{S}=\frac{\lambda^{2}I_{0}}{4\pi^{2}r^{2}}[\begin{array}{c} i_{1}(n_{r},D,\theta )\sin^{2}\varphi +i_{2}(n_{r},D,\theta )\cos^{2}\varphi ] \end{array}$$

In the equation, *I*_S_ represents the scattered light intensity of the single particle; *n*_r_ is the refractive index; *D* is the particle diameter, in meters; *λ* is the laser wavelength, in nanometers; *I*_0_ is the laser intensity, in candela; *i*_1_(*θ*) and *i*_2_(*θ*) are the scattering intensity functions in the vertical and horizontal components, respectively; *r* is the distance between the scattering point and observation point *P*, in nanometers; *θ* is the scattering angle, in degrees; *φ* is the azimuth angle, in degrees. Particle measurement techniques based on light scattering principles utilize the scattered light signals in certain direction(s) as the basis for particle size measurement, with the scattered light flux within a solid angle serving as the measurement signal [19]. The scattered light flux of particles refers to the amount of scattered light energy collected within a unit of solid angle per unit time [20]. For the calculation of particle scattered light flux, Equation (1) provides the theoretical foundation.

**Figure 2:**
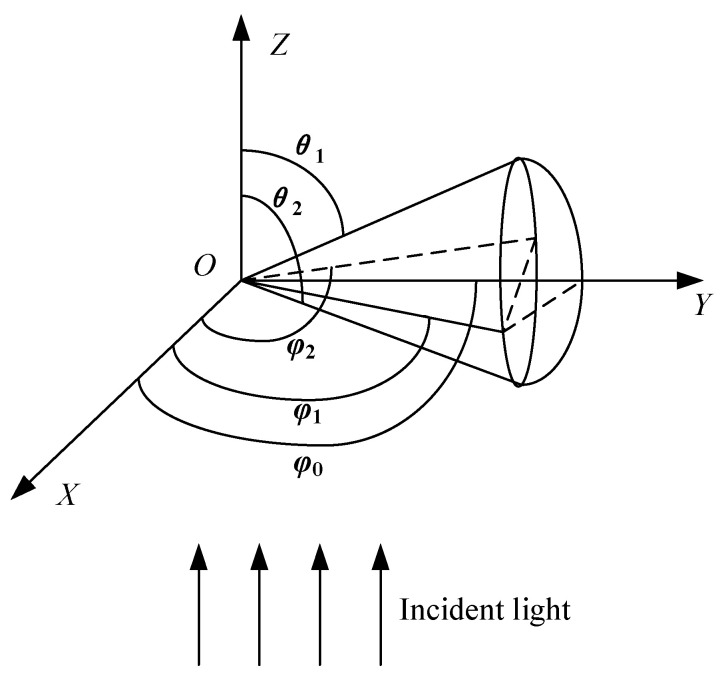
Schematic diagram of scattered light flux in the right-angle scattering light collection system.

Generally, the light scattering collection systems in optical sensors mainly have two forms: near-forward scattering collection systems and right-angle scattering collection systems [21]. The response of near-forward scattering collection systems is less influenced by the particle’s refractive index but exhibits multiple responses with changes in particle size, leading to poor monotonicity [22]. The response of right-angle scattering collection systems exhibits better monotonicity but is more significantly influenced by the particle’s refractive index [23]. However, the right-angle collection system effectively blocks stray light from the illumination system, achieving a higher signal-to-noise ratio. Therefore, this paper adopts the right-angle scattering light collection system. The schematic diagram of the scattered light flux in the right-angle scattering light collection system is shown in Figure 2, and the scattered light flux can be expressed by Equation (2).

(2) $$\begin{array}{l} F=\frac{\lambda^{2}I_{0}}{4\pi^{2}}\int_{\theta_{1}}^{\theta_{2}}\frac{i_{1}(n_{r},D,\theta )+i_{2}(n_{r},D,\theta )}{2}(\varphi_{2}-\varphi_{1})\sin\theta d\theta +\\ \frac{\lambda^{2}I_{0}\cos(2\varphi_{0})}{4\pi^{2}}\int_{\theta_{1}}^{\theta_{2}}\frac{i_{1}(n_{r},D,\theta )-i_{2}(n_{r},D,\theta )}{2}\sin(\varphi_{2}-\varphi_{1})\sin\theta d\theta \end{array}$$

here, $\varphi_{2}-\varphi_{1}=2\cos^{-1}[\frac{\cos(\frac{\theta_{2}-\theta_{1}}{2})-\cos(\frac{\theta_{1}+\theta_{2}}{2})\cdot \cos\theta }{\sin(\frac{\theta_{2}-\theta_{1}}{2})\cdot \sin\theta }]$

In the equation, *F* represents the scattered light flux in the right-angle direction, in lumens; *θ*_1_ and *θ*_2_ are the corresponding scattering angle ranges; *φ*_1_ and *φ*_2_ are the azimuth angle ranges, in degrees; *φ*_0_ is the azimuth angle of the scattering plane passing through the center of the spherical cap, in degrees. According to the research, when the refractive index of the measured particles is constant, and the scattering angle range and azimuth angle range of the instrument are fixed, the scattered light flux of spherical particles is approximately linearly related to the square of the particle diameter [24]. Since the output voltage of the photodetector is proportional to the scattered light flux of the particles, the output voltage of the photodetector also exhibits a linear relationship with the square of the particle diameter, as shown in Equation (3).

(3) $$u=cD^{2}+d$$

In the equation, *u* is the output voltage of the photodetector, in volts (V); *D* is the particle diameter, in meters (m); and *c* and *d* are constants.

The mass of a single spherical particle is given by Equation (4):

(4) $$m=\frac{\rho \pi D^{3}}{6}$$

In the equation, *m* represents the mass of a single spherical particle in micrograms (μg); *ρ* is the density of the spherical particle in micrograms per cubic meter (μg/m^3^).

Substituting Equation (3) into Equation (4) yields Equation (5):

(5) $$m=\frac{\rho \pi }{6}\cdot c^{-\frac{3}{2}}\cdot {\left(u-d\right)}^{\frac{3}{2}}$$

Therefore, if the volume and number of spherical particles with identical density and diameter are known, the corresponding mass can be calculated using Equation (5). This allows for the calculation of mass and mass concentration, as shown in Equation (6) and Equation (7), respectively:

(6) $$M=\frac{N\rho \pi }{6}\cdot c^{-\frac{3}{2}}\cdot {\left(u-d\right)}^{\frac{3}{2}}$$

In the equations, *M* represents the total mass of the spherical particle group in micrograms (μg); *N* is the number of spherical particles.

(7) $$C=\frac{M}{V}=\frac{N}{V}\cdot \frac{\rho \pi }{6}\cdot c^{-\frac{3}{2}}\cdot {\left(u-d\right)}^{\frac{3}{2}}$$

In the equation, *V* represents the volume of the unit volume of air in cubic meters (m^3^); C is the mass concentration of the spherical particle group in micrograms per cubic meter (μg/m^3^).

For ease of expressing the relationship between the mass concentration *C* and the related variables, combine the constants in Equation (7) into a single constant *K*, as shown in Equation (8):

(8) $$K=\frac{\pi }{6}\cdot c^{-\frac{1}{2}}$$

The expression for the mass concentration *C* after combining constants can be represented as Equation (9):

(9) $$C=\frac{M}{V}=K\cdot \rho \cdot {\left(u-d\right)}^{\frac{3}{2}}\cdot \frac{N}{V}$$

### 2.2. The Relationship Between Scattering Angle and Scattered Light Intensity

**Figure 3:**
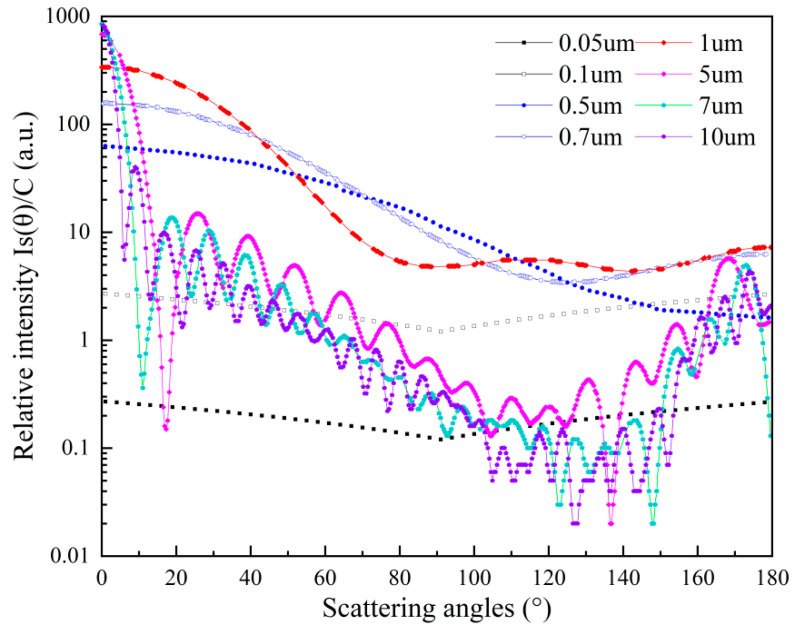
The relative intensity of scattered light from quartz particles with a mass concentration of 1 mg/m^3^, over scattering angles ranging from 0° to 180°.

Figure 3 shows the calculated curves of the relative intensity Is(θ)/C as a function of scattering angle for coal-derived aerosols within different particle size ranges, Coal-derived aerosols are more similar to diesel soot particles in terms of composition and properties, particularly their carbonaceous components and inorganic salts, which better match the characteristics of diesel soot particles. The density and refractive index of coal-derived aerosols are set to typical values of ρ = 2.6 g/cm^3^ and m = 1.5 − 0i, respectively [25]. Here, the wavelength of the incident light is 650 nm, which remains consistent throughout the experiment. The computed results are calculated according to the formula *I*s(θ)/C, where the value of *K* is simply set to 1 to obtain relative sensitivity. From Figure 3, it can be observed that for nanoparticles smaller than 0.1 μm in size, sensitivity varies very little with scattering angle, implying characteristics of Rayleigh scattering. As the size increases into the submicron range, there is often a slight oscillation. For larger particles, sensitivity varies significantly with scattering angle, rapidly decreasing in the near-forward scattering region and slowly increasing after about 90°, demonstrating oscillatory changes characteristic of Mie scattering. Additionally, for most scattering angles, the overall sensitivity of quartz particles shows a trend consistent with sensitivity in the nanoparticle range, increasing with particle size up to approximately 1 μm and then decreasing in the micron range. For particles sized 5 to 10 μm, the scattering sensitivity exhibits oscillatory changes across the range of scattering angles from 0 to 180°, with pronounced oscillations in sensitivity occurring in the near-forward scattering angles and after approximately 90°. Considering the layout of the scattering system and the variation of scattered light intensity with scattering angle, it can be concluded that the scattering system achieves better performance at a scattering angle of 90°. Therefore, this paper adopts a scattering angle of 90° for the layout of the light scattering system.

## 3. Design of the Experiment

### 3.1. Measurement System Design

**Figure 4:**
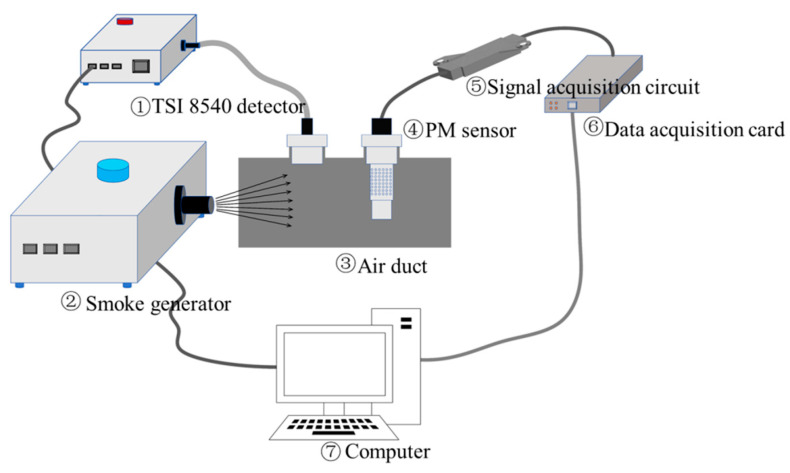
Particle mass concentration measurement system.

The particulate mass concentration measurement system is shown in Figure 4. It mainly includes the particle generation and collection module, the scattered light collection module, and the particle detection module. The particle detection module consists primarily of the TSI Dust TRAC 8540 detector (manufactured by TSI Incorporated, Shoreview, MN, USA) and a PM sensor.

The working principle of the measurement system is as follows: Particles are generated by the particle generator (in this experiment, incense sticks were used as the sample). A fan is used to evenly disperse the particles and send them into the air duct. The Dust TRAC 8540 detector and the particle sensor simultaneously draw particles from the duct at a rate of 3 L/min using a pump. The system parameters are calibrated by comparing the data directly measured by the detector with the data collected by the sensor. The scattered light signal inside the sensor’s air chamber is generated when the particles interact with the laser. After signal processing, including conversion, amplification, and filtering, the data are collected by a data acquisition card and processed in real time by the host computer. By comparing these data with the standard detector, the parameters K and d are iteratively optimized. Once the measurement accuracy requirement is met, these parameters are used for actual measurements.

**Figure 5:**
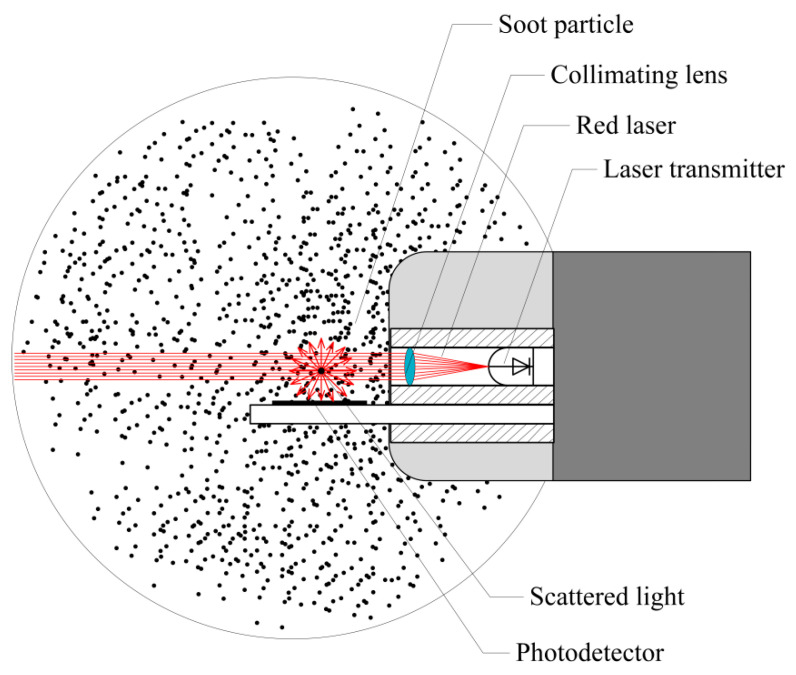
Particle scattering light collection system.

The optical scattering for the designed particulate mass concentration detection system is carried out in the scattered light collection system, as shown in Figure 5. The particles to be measured are drawn into the optical chamber at a constant speed by a vacuum pump. A semiconductor laser with a wavelength of 650 nm is directed into one side of the chamber. As the particles pass through, Mie scattering occurs. The scattered light is captured by a PIN photodetector, and the light intensity signal is converted into a voltage signal. This conversion makes the signal easier to process, transmit, and analyze.

### 3.2. Scattering Signal Analysis

**Figure 6:**
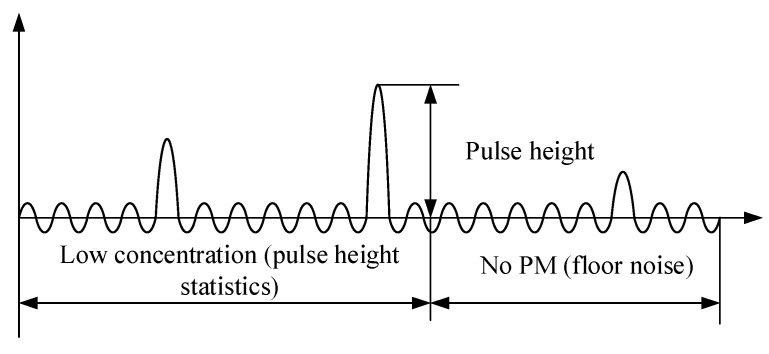
Signal detection at low concentrations.

The signal collected by the photodetector contains certain background noise, primarily originating from air molecule scattering, stray light within the optical chamber, and electrical noise. When the particle concentration is low, very few scattering signals are received, and the photodetector’s signal mostly comprises background noise, as shown in Figure 6. At this point, particles with diameters larger than the detection limit generate a series of pulse signals within the background noise. Therefore, by detecting the pulse height and count, particle size and concentration can be determined.

**Figure 7:**
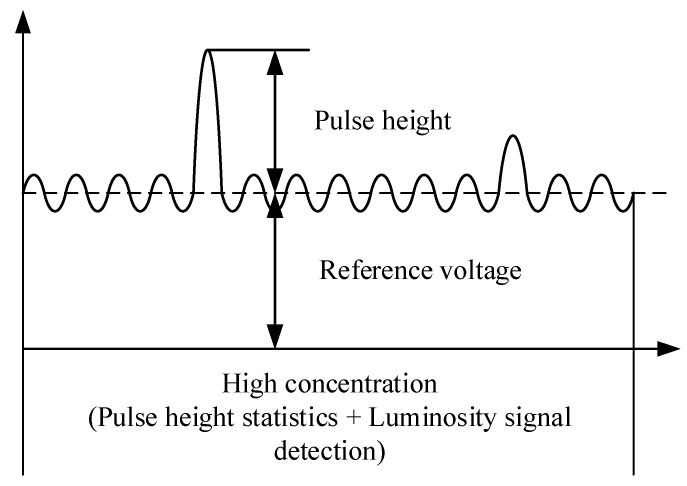
Signal detection at high concentrations.

At high concentrations, when measuring the photometric signal, smaller particles are affected by high overlap errors and may not be accurately counted, whereas larger particles can still be calibrated and calculated, as illustrated in Figure 7. The photometric response here can be defined as the light flux of aerosol scattered per unit mass concentration, reflecting particle properties such as refractive index, shape, density, and size distribution, ultimately manifested as baseline voltage. According to Mie theory, when particle size approaches the laser wavelength (650 nm), the photometric signal is most sensitive. Particles with equivalent diameters less than 1 μm will only provide a uniform amount of photometric signal, while particles with equivalent diameters of 1 to 2.5 μm will provide both photometric and pulse signals. In summary, by measuring the baseline voltage of the signal, the air quality concentration of PM2.5 can be determined.

### 3.3. Anti-Pollution Design

**Figure 8:**
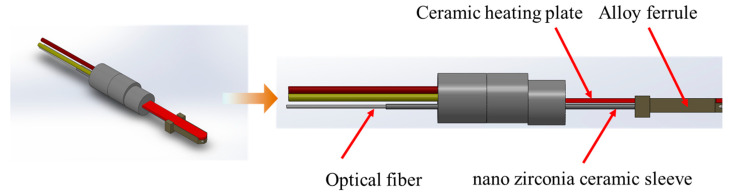
PM sensor structure.

The PM sensor needs to be installed at the back end of the DPF for long periods. After operating for some time, carbon black in the exhaust may stick to the sensor’s detector surface. This can reduce the sensor’s sensitivity and accuracy. To address this issue, we designed a structure with an alumina ceramic embedded optical fiber, combining fiber optic sensing and alumina ceramic preparation technology. The structure is shown in Figure 8.

The designed sensor integrates a simulated DPF regeneration function. The sensor’s heating control process uses dual PWM control. PWM1 is for heating control, while PWM2 is for resistance measurement. This setup allows the timely switching of control states. The platinum resistance printed on the alumina tape substrate can heat the probe to over 600 °C and maintain the temperature. During this stage, fuzzy PID control is applied to effectively reduce temperature overshoot and improve system robustness. This ensures the removal of carbon black contamination on the probe surface without affecting sensor signal detection.

In addition, the front end of the probe is fitted with a nano zirconia ceramic sleeve. Zirconia ceramic has low thermal conductivity, making it an excellent insulating material that effectively blocks heat transfer. The sleeve separates the optical fiber from the embedded ceramic heating plate’s alloy ferrule. This prevents the optical fiber from undergoing changes in optical properties due to repeated heating, which could lead to calibration drift.

**Figure 9:**
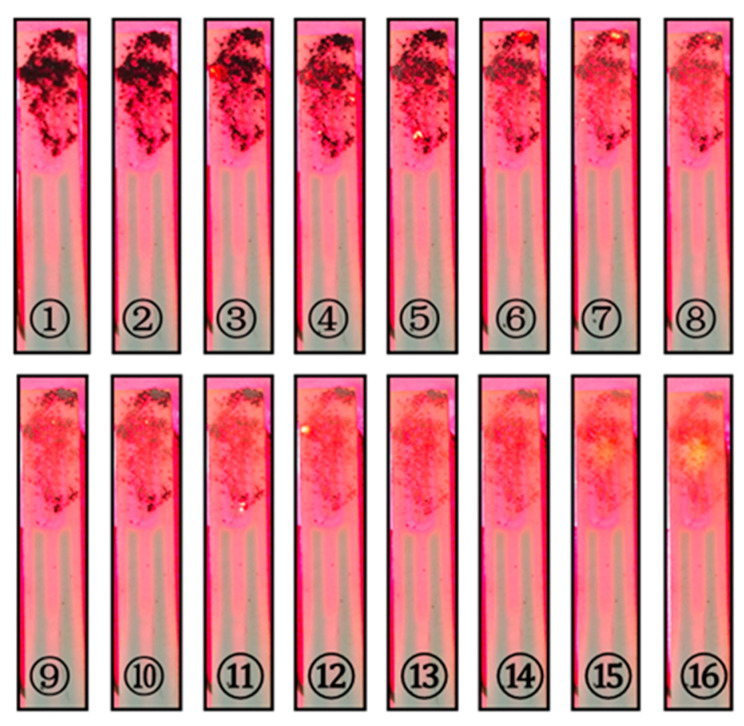
Process of removing carbon black pollution from the probe.

The carbon black on the alumina ceramic heating plate is gradually burned off during the heating process, as shown in Figure 9. In the figure, steps ① to ⑯ show the process of carbon black removal. The entire cleaning process takes only 17 s. It is clear that the designed probe structure exhibits good resistance to contamination.

## 4. Sensor Calibration and Testing

### 4.1. Calibration Experiment

Different concentrations of particulate matter were generated in a sealed test chamber. The readings from the Dust TRAK 8540 detector were used as the standard for calibration. The results obtained from the designed sensor were compared and calibrated against this standard. Throughout the experiment, the ambient temperature was maintained at 25 °C, and the chamber was free of additional light sources to prevent interference from environmental factors such as temperature and light.

**Figure 10:**
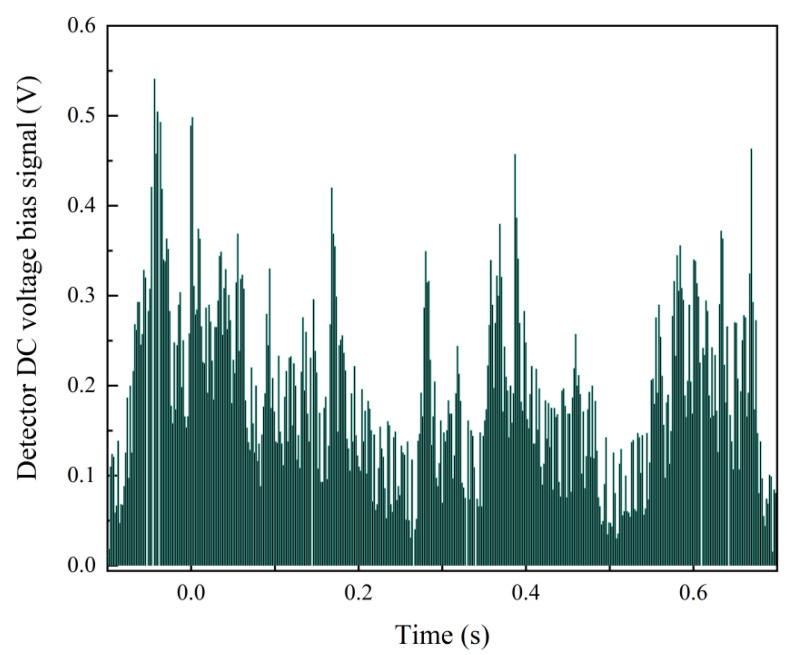
Detector DC voltage bias signal.

In practical testing, particulate matter often exhibits non-uniform shapes and varying sizes, leading to significant concentration measurement errors. Additionally, particles outside the detection limit can cause a sudden drop in photometric signals at higher ambient concentrations, as illustrated in Figure 10. This results in noticeable fluctuations in the collected voltage signals, which impede the accurate measurement of particulate matter concentration.

To address this issue, we propose a method for calculating particulate matter mass concentration based on the true RMS (root mean square) value. Typically, the average voltage signal (V_AVG_) obtained from the sensor is used as an estimate for particulate matter mass concentration. When particle size approaches 1 μm, the average value method provides a reasonably accurate estimate of the mass concentration. However, as particle size increases—especially when larger particles dominate the particle population—the average value method tends to underestimate the true mass concentration of particulate matter.

Let the voltage signal obtained from the particulate matter sensor be denoted as *V*(*t*), with a period of *T*. The signal can then be expressed as a finite Fourier series expansion:

(10) $$V(t)=a_{0}+\sum_{q=1}^{Q}[a_{q}\cos(\omega qt)+b_{q}\sin(\omega qt)]$$

here, ω = 2π*f* represents the fundamental angular frequency, where *f* = 1/*T*, *a*_0_, *a_q_*, *b_q_*, *q* = 1, 2, …, *q* is the Fourier coefficients.

(11) $$a_{0}=\frac{1}{T}\int_{0}^{T}V(t)dt=V_{AVG}$$

The corresponding true RMS value of *V*(*t*) is given by:

(12) $$V_{RMS}=\sqrt{a_{0}^{2}+\frac{1}{2}\sum_{q=1}^{Q}(a_{q}^{2}+b_{q}^{2})}$$

Consequently, the expression in Equation (13) can be obtained.

(13) $$V_{RMS}=\sqrt{V_{AVG}^{2}+\frac{1}{2}\sum_{q=1}^{Q}(a_{q}^{2}+b_{q}^{2})}$$

The true RMS value (*V_RMS_*) of the particulate matter sensor voltage signal gives greater weight to larger particles as they introduce higher amplitude pulse signals. This leads to larger values for the Fourier coefficients *a_q_* and *b_q_*. The characteristic of the true RMS method compensates for the underestimation of particulate matter concentration inherent in the traditional average value method.

**Figure 11:**
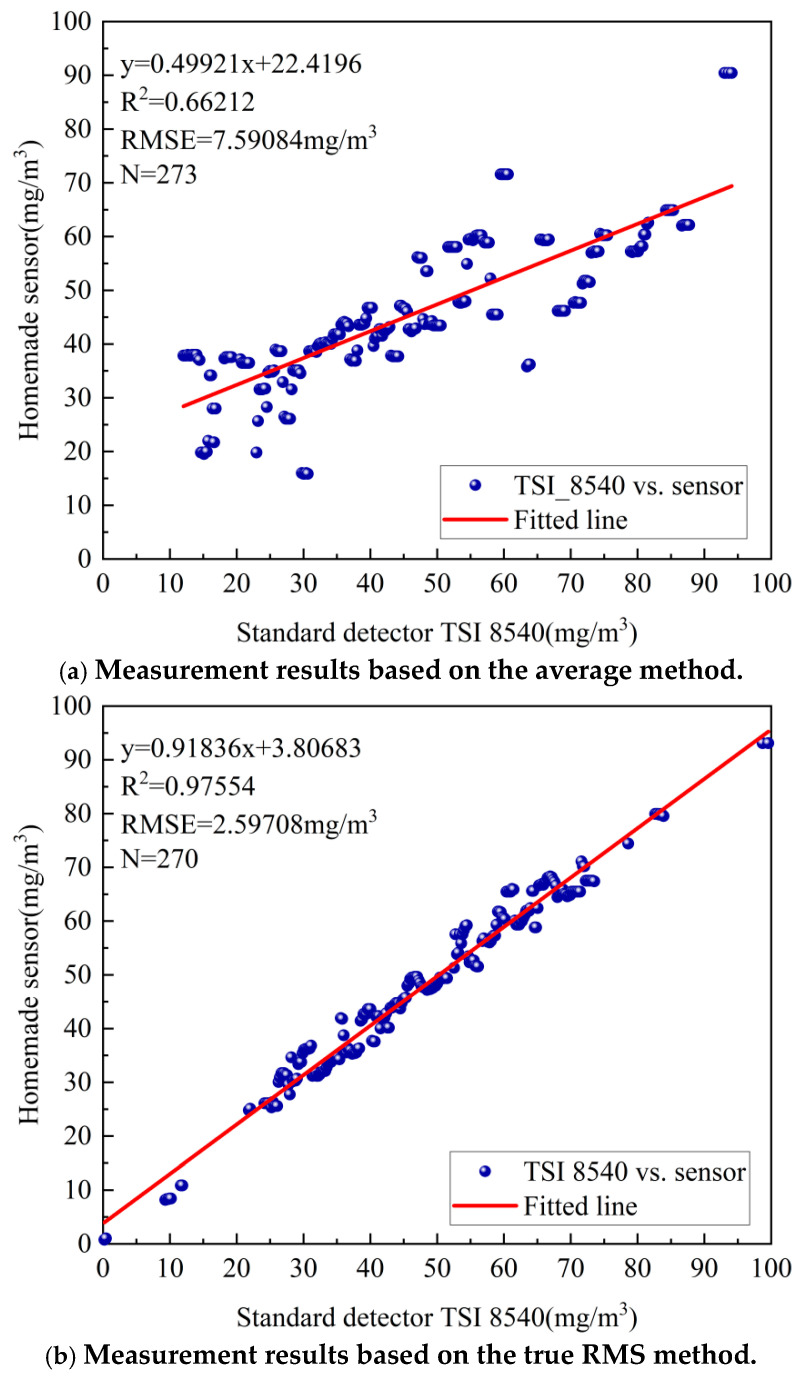
Measurement results based on average value method and true RMS method.

Figure 11a illustrates the relationship between the mass concentration obtained from the custom sensor using the average value method and the mass concentration measured by the TSI 8540 particle counter. It is evident that the correlation between the sensor’s mass concentration and the TSI 8540 readings is relatively weak, with a coefficient of determination R^2^ = 0.66212. Figure 11b presents the relationship between the mass concentration obtained from the custom sensor using the true RMS method and the TSI 8540’s readings. In this case, the correlation is significantly improved, with R^2^ = 0.97554. Furthermore, the accuracy of the particulate matter mass concentration derived from the average value method (RMSE = 7.59084 mg/m^3^) is notably lower than that obtained using the true RMS method (RMSE = 2.59708 mg/m^3^).

**Figure 12:**
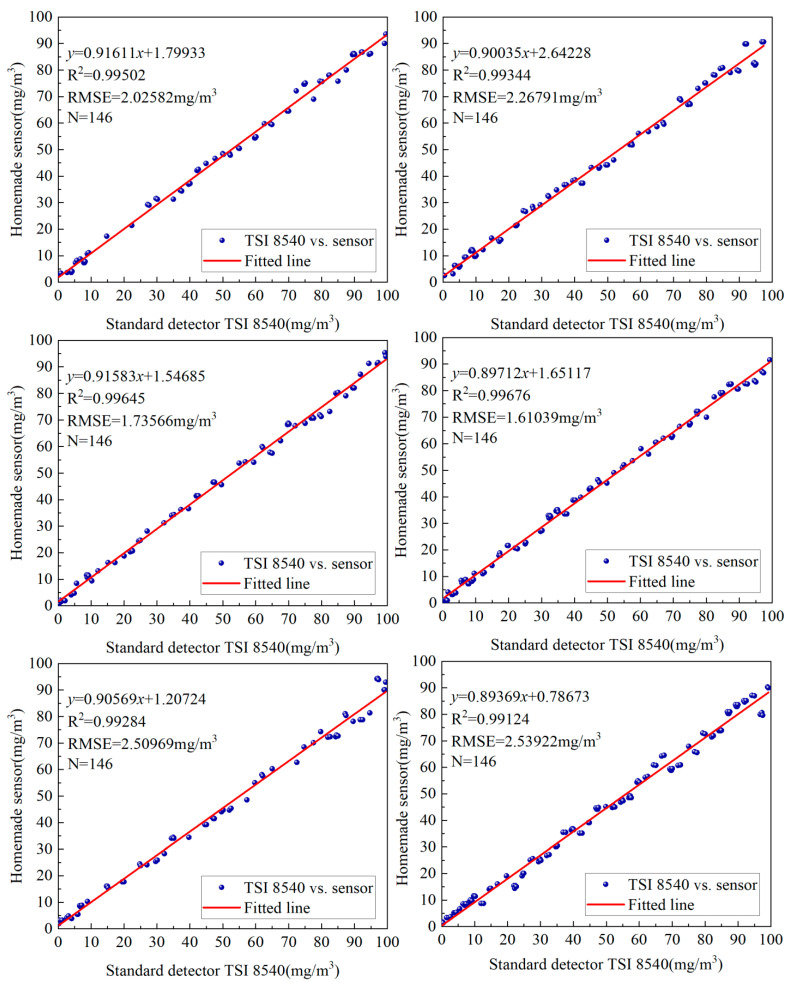
Fitted lines of six sets of experimental data.

The custom sensor, optimized using the true RMS method, was further tested in six additional experiments, all yielding favorable results. The relationship between the sensor’s readings and the data from the standard TSI 8540 particle counter is shown in Figure 12. The coefficient of determination (R^2^) representing linear correlation and the root mean square error (RMSE) indicating measurement accuracy for the six experimental sets are summarized in Table 1.

The statistical results presented in Figure 12 and Table 1 demonstrate that, after processing the particulate matter mass concentration obtained from the sensor using the true RMS method, there is a strong linear correlation between the sensor’s readings and those from the standard TSI 8540 particle counter. The coefficient of determination (R^2^) ranges from 0.99124 to 0.99676, while the root mean square error (RMSE) ranges from 1.61039 to 2.53922 mg/m^3^. These findings indicate that the true RMS method effectively mitigates the influence of large particles in complex environments, yielding highly accurate and reliable measurement results.

### 4.2. Calibration Testing

**Figure 13:**
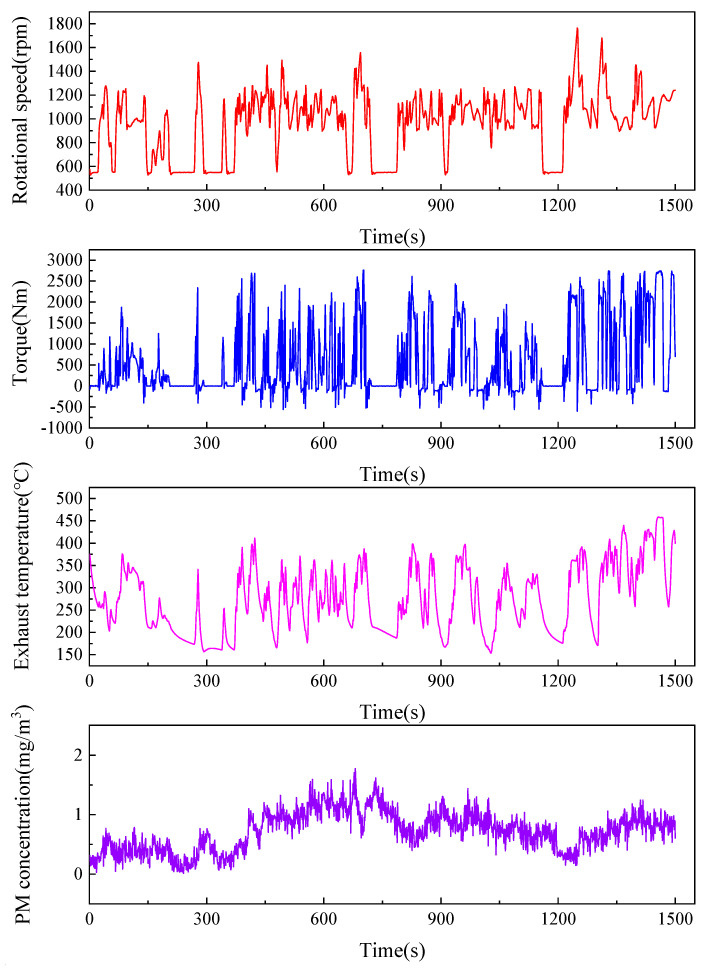
Measurement results of particulate matter emissions from engine exhaust.

**Figure 14:**
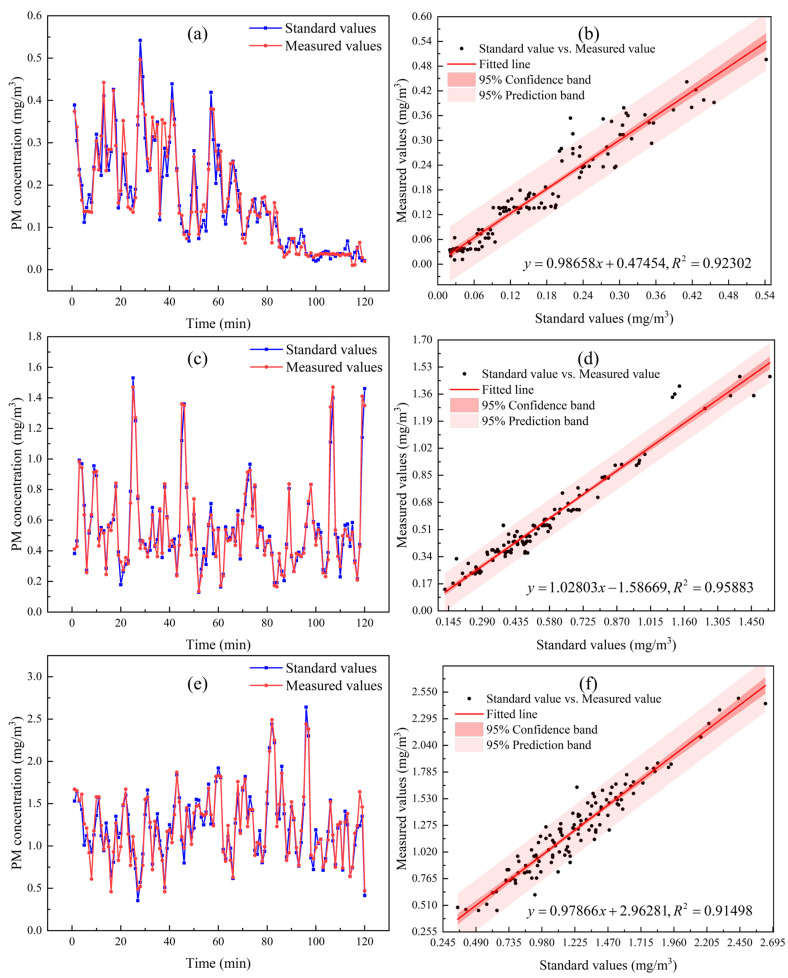
Comparison between measured values and standard values. (**a**,**c**,**e**) depict the continuous measurement curves of the corresponding sensor readings(as measured values) and standard values at different concentrations. (**b**,**d**,**f**) are linear fitting analyses of standard values and measured values in (**a**), (**c**), and (**e**), respectively.

After the aforementioned calibration process, the sensor was subjected to a continuous 120 min measurement in the same experimental environment. Sensor readings and standard values from a reference instrument were obtained and compared. Prior to the calibration test, an experiment measuring particulate matter emissions from engine exhaust was conducted, with results shown in Figure 13. The particulate matter concentration in the engine exhaust was generally maintained within the range of 0–2 mg/m^3^. Initially, the concentration was low, less than 0.2 mg/m^3^, and subsequently increased to over 0.5 mg/m^3^, with sustained fluctuations around 1 mg/m^3^. Therefore, during the calibration experiment, three sets of data were selected to represent the particulate matter mass concentration in the environment, with mean values of 0.164 mg/m^3^, 0.548 mg/m^3^, and 1.231 mg/m^3^, as shown in Figure 14. Figure 14a,c,e depict the continuous measurement curves of the corresponding sensor readings and standard values at different concentrations, while Figure 14b,d,f present the linear regression analysis of the corresponding data, yielding the fitted lines and the coefficient of determination R^2^.

Figure 14a,c,e demonstrate a strong correlation between the system’s measured values and the standard values, indicating accurate measurement of particulate matter mass concentration. In Figure 14b,d,f, the linear correlation coefficients between the two sets of data reach as high as 0.95883, confirming a high degree of correlation. These results validate the feasibility of the developed measurement system for determining particulate matter mass concentration and confirm the accuracy of its measurement outcomes.

The average relative deviations of the three sets of experimental data are 3.826%, 1.889%, and 2.128%, respectively. These results indicate that the calibrated measurement system achieves a high level of accuracy, with calibration errors well within 5%. This confirms the system’s capability to accurately measure particulate matter mass concentration.

## 5. Conclusions

This paper presents the development of a high-precision, anti-contamination optical sensor for measuring particulate matter (PM) mass concentration in diesel engine exhaust. Through calculation and analysis, it was determined that forward-angle light scattering helps reduce measurement errors in particulate concentration. Consequently, the sensor’s optical structure was further optimized. To address the issue of window contamination, an alumina ceramic structure with embedded lens fibers was designed, and dual PWM heating coupled with a fuzzy PID temperature control strategy was employed to enhance the sensor’s resistance to contamination.

By analyzing the scattering signal, the true RMS method was adopted to improve the particulate matter mass concentration measurement, ensuring the sensor’s high accuracy. Experimental results demonstrate that the developed sensor can accurately measure particulate matter concentrations with excellent stability. When the environmental concentration increased from 0.6 mg/m^3^ to 3 mg/m^3^, the relative error between the sensor’s readings and the actual concentration remained below 5%. The developed optical sensor offers a novel solution to the issue of contamination in traditional sensor probes and provides an innovative approach for monitoring the health status of automotive exhaust particulate matter filters.

## Figures and Tables

**Table 1:** Coefficient of determination and root mean square value of six sets of experimental data.

Group	Coefficient of Determination (R^2^)	Root Mean Square Error RMSE (mg/m^3^)	Number of Particles
1	0.99502	2.02582	146
2	0.99344	2.26791	146
3	0.99645	1.73566	146
4	0.99676	1.61039	146
5	0.99284	2.50969	146
6	0.99124	2.53922	146

## Data Availability

Data are contained within the article.
